# Hyperactive-impulsive behavior does not moderate the association between executive function and physical activity in preschoolers

**DOI:** 10.1038/s41598-025-96791-0

**Published:** 2025-04-21

**Authors:** Anne Eppinger Ruiz de Zarate, Julia Kerner auch Körner, Petra Haas, Catherine Gunzenhauser, Wolfgang Rauch, Caterina Gawrilow

**Affiliations:** 1 Department of Psychology, University of Tuebingen, Tübingen, Germany; 2 LEAD Graduate School & Research Network, University of Tuebingen, Tübingen, Germany; 3 IDeA Research Center for Individual Development and Adaptive Education of Children at Risk, Frankfurt am Main, Germany; 4 Institute of Psychology in Education, University of Muenster, Münster, Germany; 5 Forum of Early Childhood Education Baden-Württemberg (FFB), Stuttgart, Germany; 6 Ludwigsburg University of Education, Ludwigsburg, Germany

**Keywords:** Physical activity, Accelerometry, Executive function, Hyperactive-impulsive behavior, Preschoolers, Human behaviour, Cognitive control

## Abstract

Experimental research suggests a positive association between executive function (EF) and physical activity (PA). Observational research examining PA in everyday life does not consistently support this positive association, with findings yielding negative or no associations. Hyperactive-impulsive behavior could act as a possible moderator, explaining inconsistent findings. In this observational study, we examined the relation between EF and everyday PA as well as hyperactive-impulsive behavior as a potential moderator in a sample of 68 German preschoolers (3–5 yrs). As performance-based measured of EF and PA, participants performed a computerized EF test battery in two sessions and wore an accelerometer for 7 days. Parental questionnaires of EF, PA, and hyperactive-impulsive behavior were further implemented. Accelerometer-assessed moderate-to-vigorous PA was negatively related to EF performance, and hyperactive-impulsive behavior did not moderate this association. Neither time spent in any other PA intensity nor parental PA reports were related to EF. The present study represents the first study to investigate if hyperactive-impulsive behavior moderates the association between everyday PA and preschoolers’ EF. Thus, the findings yield new insight into the relation between PA in everyday life and preschoolers’ EF, as the unexpected negative relation could not be explained through hyperactive-impulsive behavior.

## Introduction

For a child to pursue any goal, being able to regulate their thoughts, emotions, and behavior is of central importance^1^, and executive functions (EF) play a central role for such regulation^2^. The predictive value of EF for numerous life outcomes has been repeatedly demonstrated^3,4^. The most prominent model^5^ proposes one common underlying factor with three related but distinct components: inhibition, i.e., suppressing a dominant response in favor of a less automatic action; working memory or updating, i.e., retaining and modifying of goal-relevant information; and shifting, i.e., flexible switching between mindsets as needed.

Although this three-factor structure has been frequently replicated, a recent meta-analysis^6^ suggests EF may appear more unidimensional in childhood and adolescence compared to adulthood. This shift likely reflects different developmental paths of the EF components^1^: inhibition and working memory emerge in infancy, whereas the ability for shifting develops later^7^. All three components undergo a developmental shift during preschool age, and all continue to mature through adolescence into young adulthood^8^. EF can be assessed with performance-based behavioral measures or questionnaire-based measures. Behavioral measures represent rather objective measures that aim to assess the optimal EF performance in a structured environment under favorable conditions. Questionnaire-based measures represent rather subjective measures and are better suited to capture daily EF performance in realistic and less structured environments. Accordingly, the correlations between the measurement methods are small and often non-significant^9,10^.

Because of their relevance for important life outcomes, much research has explored ways to foster EF^11^. Beside direct ways of promoting EF, for example through computerized programs, other programs aim to indirectly promote EF, for example by increasing physical activity (PA)^12^.

### Executive function and physical activity

PA refers to any bodily activity that increases an individual’s energy expenditure, and it is characterized by its frequency, duration, type, and setting^13^. Numerous findings from experimental studies consistently support a positive effect of PA on EF in older adults^14,15^, whereas there are fewer and less consistent empirical results for children^16^—especially in preschoolers^17^. Given the fast developmental changes in preschoolers, findings from older age groups may not be transferrable^18^.

Moreover, findings might differ depending on the type of PA investigated. A meta-analysis of ten experimental studies revealed small to moderate positive effects of PA interventions with a duration of at least 4 weeks on preschoolers’ EF^18^. However, experimental findings do not necessarily translate to the association of PA in everyday life with EF. Observational studies examine everyday PA—independent of specific PA interventions. This is especially interesting to better understand how a physically active lifestyle influences EF performance and development. In a meta-analysis^17^, six of seven studies in early childhood (i.e., 5 experimental, 2 observational) reported a positive relation between PA and EF. The authors highlighted that none of the reviewed studies reported negative effects of PA on EF, and the positive association was also evident in the observational studies.

PA can be assessed with behavioral measures like accelerometers or with questionnaire-based measures. Only a limited number of observational studies have employed accelerometers as a direct and valid measure of PA^17^. This is important to note, because (a) correlations between accelerometer- and questionnaire-assessed PA levels are low-to-moderate^19^, and (b) it is not yet fully understood how aspects of PA quantity are related to EF in preschoolers^20^. Accelerometers are well-suited to examine PA quantity. An often-examined aspect of quantity is time spent in different PA intensities, ranging from light PA (e.g., walking below 4 km/h) to moderate PA (e.g., walking with 4–7 km/h) and vigorous PA (e.g., running with 7 km/h). In the literature, the most frequently examined intensity is moderate-to-vigorous PA (MVPA). The PA guidelines by the World Health Organization (WHO) recommend that averaged across 1 week, children should undertake 60 min per day of MVPA.

An observational study reported that seven-day MVPA but no other PA intensity was positively related to inhibition^21^. Yet, recent studies in preschool samples reported negative associations between PA and EF: One study reported positive effects on EF when reallocating as little as five minutes spent in sleep or light PA to time spent in MVPA^22^. However, the direct association between MVPA and EF in the same sample was negative. Another study supported this negative direct association between MVPA and preschoolers’ EF^23^. However, both studies included preschool samples with low socioeconomic status from Brazil^22^ or rural South Africa^23^. The latter sample exhibited generally high levels of MVPA but low levels of structured PA. The authors argue that the combination of high levels of naturally occurring unstructured PA (e.g., running around) and low levels of structured PA (e.g., teacher-arranged) might not necessarily enhance EF development. Thus, these findings might not be generalizable to samples with high socioeconomic background and potentially higher levels of structured PA. In line with this argument, in a large Australian cohort sample with high socioeconomic background, most preschoolers participated regularly in structured PA (e.g., team sports), and participation in structured PA was related to better shifting performance^24^. In the same sample, naturally occurring MVPA measured with an accelerometer over 7 days was not related to EF^24^. These findings suggest that inconsistent findings might possibly be explained by the implemented study design and PA measurement method.

One study employed the same measurement methods as the present study to investigate the association between everyday PA and EF in preschoolers: Willoughby et al.^25^ examined a sample of 85 US preschoolers that completed the test battery to assess EF performance and wore accelerometers for 5 weekdays to measure naturally occurring everyday PA. Contradicting their expectations, MVPA was negatively related to overall EF and to inhibition when controlling for child gender, age, Body-Mass-Index (BMI), and parental level of education. As a possible explanation for the unexpected negative results, Willoughby et al. discussed the idea that hyperactive-impulsive behavior might be related to PA such that hyperactive-impulsive children spend more time in higher intensity PA in unstructured settings^26^.

### The role of hyperactive-impulsive behavior

Hyperactive-impulsive behavior includes motor restlessness and high levels of unstructured PA. Further, it is one of the main symptoms of attention deficit hyperactivity disorder (ADHD)^27^, a form of externalizing disorders closely linked to EF deficits^28^. Symptoms of ADHD can already emerge in preschoolers, but ADHD is typically diagnosed later in childhood^29^. Yet, findings from older ADHD populations can yield important insights into the role of hyperactivity for the association between PA and EF in preschoolers.

The role of PA—a key component of hyperactivity—for ADHD is still discussed in the literature: Eminent theoretical models view hyperactivity as a core symptom and, thus, a core diagnostic feature of ADHD. Accordingly, hyperactivity is seen as ubiquitous and a reflection of a deficit to inhibit task-irrelevant behavior^28^. Thus, hyperactivity represents a result of EF deficits in ADHD populations. Previous research supports differences in PA levels between children with ADHD and the general population^30,31^, and parent-reported hyperactivity is related to higher objectively measured PA levels in clinical^32^ and non-clinical samples^33^. Accordingly, and in line with Willoughby et al.’s^25^ argument, MVPA was related to more externalizing problems in a sample of preschoolers^34^.

An alternative scientific narrative views hyperactivity as a compensatory mechanism facilitating cognitive functioning in ADHD populations^35^. Experimental studies in older children and adolescents with ADHD suggest that PA interventions are beneficial for EF^36^. PA levels in childhood predict ADHD symptomatology in adolescence^37^, and a longitudinal nation-wide German survey showed that MVPA in preschool age might be specifically beneficial for the EF development across childhood^38^. For instance, MVPA was associated with favorable changes in ADHD-symptomatology across 1 year in preschoolers with lower EF capacity and possibly at-risk for ADHD^39^.

Evidence further supports that hyperactivity functions as a compensatory mechanism during tasks with high EF demands: children with ADHD compensate for lower EF capacities with higher levels of PA. A meta-analysis of 63 studies suggests that PA levels in ADHD populations increase during high EF demand settings^32^. Evidence suggests that PA during cognitive tasks is positively related to inhibition^40^ and working memory^41^ in older children with ADHD but not in typically developing peers. This effect has not been reported for shifting^42^.

First evidence from a sample of non-clinical but at-risk younger children (aged 5–7 yrs) with early ADHD symptoms also supported an increase in PA levels during a task with high EF load^33^. Elevated PA levels during an inhibition task were related to lower task performance only in preschoolers with high hyperactivity symptoms. This suggests that hyperactive-impulsive behavior moderates the relation between PA and EF in young children. However, it is not clear whether and how these findings can be transferred to PA exhibited in everyday life. It is not yet definite whether everyday PA may be positively related to EF in children with higher levels of hyperactive-impulsive behavior^39,43^ or in children with lower levels^25^. Further research examining the potential moderating role of hyperactive-impulsive behavior on the association between everyday PA and EF in preschoolers is necessary.

### Present research

The central aim of our study was to examine the association between everyday PA and EF in preschool children. For this, we conducted a study with 68 preschoolers that completed the German version of the EF Touch^44^, a highly differentiated and reliable test battery to assess preschoolers’ EF. We measured their PA with an accelerometer for seven consecutive days. Further, we implemented parental questionnaires as rather subjective measures of EF and PA.

The focus of the present study was on the behavioral measures of EF and PA, which promise a higher objectivity but capture only a selective performance over a limited time. To ensure the validity of the behavioral measures, we examined the association to the questionnaire-based measures, which are more subjective but reflect a longer observation period. We expected a positive association between behavioral and questionnaire-based measures for both PA (H1.1) and EF (H1.2).

For the further hypotheses, we focused on the behavioral measures of PA and EF: Since observational findings suggest a positive association between PA and EF in older samples and some also support this in preschoolers, we expected a positive relation between MVPA and EF (H2.1). Additionally, we hypothesized that controlling for hyperactive-impulsive behavior would improve the prediction of EF (H2.2).

Another aim of our study was to investigate Willoughby et al.’s^25^ assumption that hyperactive-impulsive behavior could explain the negative association between MVPA and EF. To our best knowledge, the present study is the first observational study to investigate whether hyperactive-impulsive behavior moderates the association between behavioral measures of everyday PA and EF in preschoolers. We hypothesized that hyperactive-impulsive behavior would moderate the relation between MVPA and EF (H3). As the direction of the influence is not yet definite^25,39,43^, we formulated a non-directional hypothesis.

In explorative analyses, we further examined (1) if MVPA would be differently related to the specific EF components (i.e., inhibition, working memory, shifting), and (2) if time spent in distinct PA intensities (i.e., light, moderate, vigorous) would be differently related to total EF performance. Because of the limited previous evidence for specific EF components and PA intensities, we formulated non-directional explorative hypotheses. Additionally, if behavioral and questionnaire-assessed measures of PA were not at least moderately related, we wanted to examine (3) if parent-reported PA was positively related to total EF performance in the EF Touch.

## Methods

### Procedure and participants

This study was part of a wider project aiming to standardize and validate the German version of the EF Touch test battery (*ExFunKi*—*Exekutive Funktionen im Kindergarten*). It was approved by the ethics committee of the German Psychological Society (KörnerJulia2020-01-28WV), and all methods were performed in accordance with the relevant guidelines and regulations. In total, data was collected from 442 German preschoolers (aged 3–5 yrs) across four regions (Hamburg, Tübingen, Leipzig, Koblenz-Landau) between 2021 and 2023, with procedures and implemented measures varying between sub-samples. All children completed the German version of the EF Touch Test battery in the same order as well as tasks to evaluate their cognitive and language development. Testing took place at a laboratory or in kindergartens. Parents filled out questionnaires assessing sociodemographic data as well as EF and temperament among others. If the testing took place in the kindergarten, kindergarten teachers were also asked to fill out questionnaires assessing the same constructs. For the validation, stratified sub-samples were assigned to following conditions: (a) baseline with only EF Touch and questionnaires, (b) retest condition after approximately 14 days, (c) additional behavioral EF measure, (d) delay of gratification task, (e) intelligence test, (f) test of mathematical competences, or (g) accelerometers. These conditions could also be mixed (e.g., accelerometer and retest condition). An extensive description of the project can be found in the study protocol (in prep., Kerner auch Körner et al.). Age between 3 and 5 years was the only eligibility criterion.

For the present study, we only used data of the specific sub-sample wearing an accelerometer and will therefore only describe the relevant procedure and measures. In this sub-sample, preschoolers wore an accelerometer for seven consecutive days, measuring their everyday PA. We received written consent for 80 children. In total, 12 children were excluded from analyses due either to insufficient PA data recording with the accelerometer (*n* = 5), missing data in the EF tasks (*n* = 1), insufficient understanding of the EF tasks (*n* = 1), or missing data on hyperactive-impulsive behavior (*n* = 5).

Thus, the final sample size consisted of 68 preschoolers, with an equal number of female (*n* = 34) and male (*n* = 34) participants. On average, participants were aged 56.41 months (*SD* = 8.91 mths; range 36.26–71.81 mths) and had an average BMI of 15.39 (*SD* = 1.68; missing *n* = 13). Regarding the parental level of education, 72.06% of families showed high, 16.18% average, and 10.29% low levels (for classification see Ulitzka et al.^44^). This sample was recruited in regional kindergartens and through university-wide e-mails at the University of Tübingen (Southwestern Germany; *n* = 55) and the University of the Federal Armed Forces Hamburg (Northern Germany; *n* = 13). After receiving parental written consent, preschoolers were individually tested by one instructor in their kindergarten (*n* = 42) or in the laboratory at the university (*n* = 22) at two separate appointments; these could either be scheduled on the same day with a break (*n* = 8) or on two different days (*n* = 58). We observed no significant difference in the EF performance (*z*(*N1* = 42, *N2* = 22) = 461.5; *p* = 0.397) or in time spent in MVPA (*z*(*N* = 42, *N2* = 22) = 571; *p* = 0.588) depending on the testing site. There was no difference in EF performance (*z*(*N1* = 8, *N2* = 58) = 288; *p* = 0.276) or time spent in MVPA (*z*(*N1* = 8, *N2* = 58) = 217; *p* = 0.776) between children performing the EF Touch on 1 day or 2 days.

At the start of the first appointment, preschoolers were informed in an age-appropriate way about the experiment and the accelerometer measurement, they were asked for their verbal consent and were shown how to wear the accelerometer. Afterwards, they completed the first part of the computerized EF tasks (i.e., EF Touch), consisting of a short training task and the first five EF Touch subtests. In the second appointment, preschoolers completed the remaining two EF Touch subtests. The EF Touch tasks were always presented in the same order (see Table 1). After completing the EF Touch, the investigator measured the children’s height and weight.

**Table 1:** Description of the EF touch tasks, including the correspondent executive function component measured.

Task	Executive function component	Task description	Items	Items for 3-year-olds	RT measurement	Data exclusion
First appointment						
Something’s the same	Shifting	Recognize similarities between pictures on three dimensions (color, size, shape)	30	20	No	4^*a*^
Arrows	Inhibition	Press button in direction that arrow points at (congruent, incongruent trials)	36	All	Yes	3
Houses	Working memory	Remember animal(s) and color(s) of house(s)	18	0	No	4
Pig (Go/NoG)	Inhibition	Press button when animals are presented; except for pig	40	All	Yes	4
Farmer	Working memory	Remember movements of animals across a 4 × 4 matrix	36	8	Yes	3
Second appointment						
Pick the picture	Working memory	Select each picture once; position of pictures rotates	32	All	No	4
Silly sounds game (stroop)	Inhibition	Press cat when hearing bark, press dog when hearing meow	17	All	Yes	2

EF Touch subtests were always presented in the order depicted above. Items for 3-year-olds describe the number of items presented in this age group; some items were omitted because of increased task difficulty. Data exclusion specifies the number of children for which data from the task was excluded because of problems in the task administration (e.g., external disturbance) or limited understanding of the task/instruction.

^a^For two additional children, data was excluded for the second part of the task because of limited understanding.

Participation was voluntary, and preschoolers received a small present as well as a certificate after each appointment. Parents received information about their child’s PA, and kindergartens were rewarded with a book about EF as well as an optional workshop.

### Material

#### Executive function

##### EF touch

As a behavioral measure of preschoolers’ EF, we implemented the German version of the EF Touch^44^, originally developed by Willoughby and Blair^45^. The EF Touch is a computerized test battery conducted on a 15-inch touch screen, consisting of one training task measuring reaction time (RT) and seven age-specific tasks measuring EF. Regarding the separate EF components, three tasks measured inhibition, three working memory, and one task measured shifting. **S**hort descriptions of each task can be found in Table 1. After each task, instructors indicated the child’s comprehension on a 10-point Likert-scale (*How well did the child understand the task?* from *1*—*not at all* to *10*—*very well understood*) and noted any further comments in an open format.

In line with previous analyses of the German EF Touch^44^, we excluded trials with RT of less than 200 ms in tasks with RT measurement. As indicated in the pre-registration (see: https://osf.io/uc8vn), for trials with RT deviating more than 1.5 of the interquartile range from the individual mean RT, we inspected investigators’ answers to the protocol question about the child’s understanding as well as further comments. If investigators indicated problems with a child’s comprehension in the protocol question (scores 1–3) or problems in test application (e.g., external disturbance), data from the respective tasks was excluded from the analysis (see Table 1). Deviating from the pre-registered analysis plan, we inspected investigators’ comments and excluded data equivalently in trials without RT measurement.

After data exclusion, we calculated accuracy scores (1–100%) for each EF Touch task. Ulitzka et al.^44^ found that the tasks sufficiently differentiate between the three EF components, but because of high interrelations between the components the one-factor EF model represented the data better. Thus, we computed a total EF Touch score averaging the accuracy scores of all tasks to capture children’s EF competence. For the exploratory analyses examining the single EF components (i.e., inhibition, working memory, shifting), we calculated the average accuracy across the respective EF Touch tasks.

##### BRIEF-P

As a questionnaire-based measure of EF, we employed the German version of the *Behavior Rating Inventory of Executive Function*—*Preschool Version* (BRIEF-P)^46^, consisting of 63 items. Parents rated how often their child’s exhibited behaviors presented a problem during the last 6 months (e.g., *Was impulsive* or *Needs support from adults to stay focused on one task*) on a 3-point scale (*‘never’*, *‘sometimes’*, or *‘often’*). We calculated the mean score for all answers (range 1–3), with higher values implying lower executive function. The scale showed a very high reliability *α* = 0.96.

#### Physical activity

##### Accelerometer

As a behavioral measure of PA, participants wore an Actigraph GT3X + (Actigraph, LLC, Fort Walton Beach, Florida). After the first appointment, participants wore the accelerometer during waking hours on their hip on the non-dominant side for seven consecutive days, measuring PA in 15 s epochs.

The analysis of accelerometer data followed established guidelines regarding data exclusion, valid wear time, and cut-points: To categorize different PA intensities, we applied the age-specific cut-points defined by Pate et al.^47^ for the vertical axis 1, shown to most accurately classify MVPA^48^ and previously employed in similar work^25^. We classified 20 min of consecutive zeros as non-wear time. Days with at least 6 h of wear-time were defined as valid days, and children with at least three valid days were included in the analysis^49^. We calculated the proportional time spent in each PA intensity (MVPA, light, moderate, vigorous, sedentary) by dividing the minutes spent in the correspondent intensity^47^ across the 7 days through the total minutes of collected PA data.

##### PA questionnaire

We also implemented a short parental questionnaire to assess PA^50^. Parents described their child’s regular PA by answering seven items on 5-point scales. The scales differed between items: The item *Does your child attend a sports club or sports group?* was answered on scale ranging from *1*—*no* to *5*—*regularly more than 2 h per week*. The item *How often do you or others go to the public pool with your child?* was answered on a scale ranging from *1*—*never* to *5*—*often*. The remaining 5 items (i.e., climbing, playing ball, play catch, ride bike or scooter, roller skating) were answered on a scale ranging from *1*—*never* to *5*—*every day*.

In line with the original validation study^50^, answers were defined as high (+ 1) or low (− 1) levels of PA. As suggested in previous work with this questionnaire^51^, we calculated the sum score of all answers (− 7 to 7) as a continuous measure of PA. In our sample, the scale showed a very poor reliability *α* = 0.36. We argue that the questionnaire is still interpretable regards content and theoretical background despite the low numerical alpha value^52^: the PA questionnaire assesses the frequency of different types of PA (e.g., playing with a ball, attending a sports club), which can occur independent of each another. Thus, to discriminate and assess these distinct types of PA, high alpha values are not necessarily desirable^53^. Still, the low reliability needs to be considered when interpreting the results, as it might limit the possibility of detecting associations with other measures.

#### Hyperactive-impulsive behavior

Hyperactive-impulsive behavior was measured using the parent-report version of the German translation of the *Strengths and Difficulties Questionnaire for Preschoolers* (SDQ 2–4)^54^. We calculated the mean score of the respective scale *Hyperactivity*, including 5 items (e.g., easily distracted, concentration wanders; thinks things out before acting), comprising all three symptom categories of ADHD (i.e., hyperactivity, impulsivity, inattention). The items for the hyperactivity scale are the same in the version for 2–4-year-olds and in the version for the older children. Parents rated their child on a 3-point Likert-scale (*1*—*not applicable* to *3*—*clearly applicable*), with higher values implying more hyperactive-impulsive behavior. The scale showed a very good reliability *α* = 0.83.

#### Background measures

Parents answered questions about their children’s age and gender, as well as level of education for both parents on a five-point scale ranging from *1*—*No high school graduation* to *5*—*University-entrance diploma [“Abitur”]*. In line with previous analyses^44^, the level of education of both parents was averaged and then rounded down. If level of education was just indicated for one parent, this value was used as a proxy. Further, to calculate children’s Body-Mass-Index (BMI), investigators measured children’s height and weight.

### Data analysis

Analyses were pre-registered (see: https://osf.io/uc8vn) and conducted with R version 4.3.2 (R Core Team, 2023), using *α* = 0.05 to denote statistical significance. Regarding data preparation of the EF Touch, we had only pre-registered data exclusion based on investigator’s comments for the tasks with RT measurement. However, in addition to the pre-registration, we conducted a parallel data exclusion for tasks without RT measurement. In contrast to the accuracy score computed by the EF Touch^45^, which is based on the number of items a child sees, in line with Ulitzka et al.^44^ we computed the accuracy based on the total number of items of each task.

We tested the association between behavioral and questionnaire-based measures of PA (H1.1) as well as EF (H1.2) with two separate correlation tests. For the following analyses, we used the behavioral measures for EF and PA: To test the Hypotheses H2.1 and H2.2 regarding the association between PA and EF, we used linear regression models with total EF performance as outcome and MVPA as predictor. In Model 1 (H2.1), we controlled for age, child gender, BMI, and parental level of education, and in Model 2 (H2.2), we additionally controlled for hyperactive-impulsive behavior. All control variables and the predictor were centered on the grand mean. To test if hyperactive-behavior moderated the relation between MVPA and total EF (H3), we included an interaction term between MVPA and hyperactive-impulsive behavior in Model 3, multiplying the respective variables. Normality tests were conducted for PA and EF data. For all linear regression models, we further checked for linearity, homoscedasticity, and outliers to ensure model assumptions were not violated. Outliers were excluded, and in cases of heteroscedasticity we calculated Weighted Least Squares to account for unequal variance of observations.

In the exploratory analyses, we again conducted linear regression models to examine: (1) the relation between MVPA and the separate EF components (Exporative Analysis 1), (2) the relation between time spent in different PA intensities and total EF Touch score (Explorative Analysis 2), and (3) the relation between parent-reported PA level and total EF performance. The models for the explorative analyses are described in the Supplementary Material.

## Results

### Descriptive results

Data availability as well as descriptive results of accelerometer and EF Touch data are summarized in Table 2. For more than half of the sample (63.24%), accelerometer data was available for 7 days with an average of 10 h of data collection per day (*M* = 10.04, *SD* = 2.50). During the data collection period, children spent approximately 51% of the time in sedentary behavior, 36% in light PA, and 13% in MVPA. On average, participants spent approximately 80 min per day in MVPA, fulfilling the WHO guideline. In the EF Touch, children on average showed a total EF Touch score of 72.02% (range 23.20–90.01).

**Table 2:** Description of objective data for physical activity (accelerometer) and executive function (EF touch).

Variable	Metric	*N*	*M (SD)*
Accelerometer	Number of valid days (max. 7)	68	6.69 (1.33)
	Total wear-time (hrs/day)	68	10.04 (2.50)
Daily activity level^a^	Sedentary (mins/day)	68	307.8 (97.46)
	Light PA (mins/day)	68	214.76 (59.24)
	MVPA (mins/day)	68	79.86 (30.43)
Executive function	Total executive function (% correct)	68	70.39 (12.68)
	Inhibition (% correct)	67	82.34 (16.10)
	Working memory (% correct)	66	57.45 (14.02)
	Shifting (% correct)	65	73.79 (18.31)

*MVPA* moderate-to-vigorous physical activity.

^a^Daily Activity Level refers to the time spent in each physical activity level per day measured with the accelerometer.

In the parental questionnaires, overall parents described their children’s PA level as intermediate (*M* = 0.45, *SD* = 2.91) and EF capacity as medium (*M* = 1.57, *SD* = 0.33). They described children’s hyperactive-impulsive behavior as relatively low (*M* = 0.65) and the variability in the sample was also low (*SD* = 0.50). The bivariate associations between all assessed variables are presented in a correlation matrix in the Supplementary Material (Supplementary Table S1).

### Hypothesis testing

The correlation tests examining the association between behavioral and questionnaire-based PA measures (H1.1) revealed no significant correlation between accelerometer-assessed MVPA and parental-reported PA (*r* = 0.005, *t*(65) = 0.040, *p* = 0.968). Regarding the association between behavioral and questionnaire-based EF measures (H1.2), total EF Touch score and parental reports in the BRIEF-P were significantly correlated (*r* = − 0.248, *t*(66) =  − 2.080, *p* = 0.041): Higher total EF Touch score was related to less parent-reported EF-related problems. Considering that the questionnaire assessed EF-related problems while the EF Touch measured EF performance, this result pattern matches the hypothesized direction.

To test the association between accelerometer-assessed MVPA and total EF Touch score (H2), we ran two multiple linear regression models, displayed in Table 3*.* In Model 1 testing H2.1, we controlled for possible confounders (i.e., age, child gender, BMI, parental level of education), and in Model 2 testing H2.2, we additionally controlled for hyperactive-impulsive behavior. As Model 2 did not predict the data significantly better than Model 1 (*F*(1, 58) = 1.286, *p* = 0.262, f^2^ = − 022), controlling for hyperactive-impulsive behavior did not significantly improve the prediction of total EF Touch score. Accordingly, we used Model 1 to investigate the relation between MVPA and EF: more time spent in MVPA was related to significantly lower total EF Touch score (*β* = − 0.55, *p* = 0.038). Further, age (*β* = 0.70, *p* < 0.001) and parental level of education (*β* = 2.40, *p* = 0.009) were related to better EF performance.

**Table 3:** Relation between MVPA and executive function performance (Hypothesis 2) with possible moderating effect of hyperactive-impulsive behavior (Hypothesis 3).

	Model 1			Model 2			Model 3		
	Est	CI	*p*	Est	CI	*p*	Est	CI	*p*
Intercept	**60.59**	**52.71–68.47**	** < 0.001**	**61.06**	**53.15–68.97**	** < 0.001**	**60.92**	**52.95–68.89**	** < 0.001**
MVPA [in %]	**− 0.55**	**− 1.07 to − 0.03**	**0.038**	**− 0.60**	**− 1.12 to − 0.07**	**0.027**	**− 0.65**	**− 1.21 to − 0.09**	**0.023**
Age	**0.70**	**0.48–0.93**	** < 0.001**	**0.68**	**0.46–0.91**	** < 0.001**	**0.71**	**0.48–0.94**	** < 0.001**
Gender (female)	0.93	− 2.74 to 4.60	0.614	0.47	− 3.28 to 4.22	0.801	0.45	− 3.42 to 4.33	0.815
Body Mass Index	− 0.72	− 1.92 to 0.47	0.230	− 0.59	− 1.80 to 0.62	0.335	− 0.54	− 1.76 to 0.69	0.383
Level of parental education	**2.40**	**0.62–4.17**	**0.009**	**2.33**	**0.55–4.10**	**0.011**	**2.39**	**0.59–4.20**	**0.010**
Hyperactivity/impulsivity				− 2.17	− 6.00 to 1.66	0.261	− 1.74	− 5.82 to 2.33	0.396
MVPA X hyperactivity/impulsivity							0.33	− 1.07 to 1.73	0.638
*N*	65			65			64		
R^2^	0.583			0.592			0.602		
Adjusted R^2^	0.548			0.550			0.552		

Executive Function Performance is operationalized as the overall accuracy score in the EF Touch test battery; MVPA = moderate-to-vigorous physical activity; age was calculated in months; MVPA X Hyperactivity/Impulsivity = interaction between MVPA and hyperactive-impulsive behavior; Hyperactive-impulsive behavior was assessed through a parental questionnaire.

Significant values are in bold.

To examine whether hyperactive-impulsive behavior possibly moderated the relation between MVPA and EF (H3), we added the interaction term between MVPA and hyperactive-impulsive behavior in Model 3. Results can be inspected in Table 3. Again, time spent in MVPA was significantly and negatively related to total EF Touch score (*β* = − 0.65, *p* = 0.023). The interaction between MVPA and hyperactive-impulsive behavior was not significant (*β* = 0.33, *p* = 0.638); thus, hyperactive-impulsive behavior did not moderate the association between MVPA and total EF Touch score. Concerning the confounding variables, age (*β* = 0.71, *p* < 0.001) and parental level of education (*β* = 2.39, *p* = 0.010) were related to better EF performance.

### Explorative analyses

Here, we will briefly present the key findings of our explorative analyses. The models testing the explorative hypotheses and the results are provided in detail in the Supplementary Material.

Explorative Analysis 1 revealed that there was no significant association with MVPA and the separate EF components when controlling for possible confounders (i.e., age, gender, BMI, parental education, hyperactive-impulsive behavior; inhibition: *β* = − 0.32, *p* = 0.303; working memory: *β* = − 0.46, *p* = 0.235; shifting *β* = -0.03, *p* = 0.941). In Explorative Analysis 2, we separately examined different PA intensities and found no significant relation to total EF Touch score (light PA: *β* = − 0.14, *p* = 0.558; moderate PA: *β* = 0.07, *p* = 0.934; vigorous PA: *β* = − 1.45, *p* = 0.137). Lastly, parent-reported PA was also not related to total EF Touch score (*β* = − 0.25, *p* = 0.424).

## Discussion

In our observational study with 68 German preschoolers, we examined how PA in everyday life is related to EF and whether hyperactive-impulsive behavior influences this relation. To our best knowledge, the present study is the first study to examine a potential moderation effect of hyperactive-impulsive behavior on the relation between PA and EF in preschoolers. We focused on the age group of preschoolers (aged 3–5 yrs) because of the fast developmental changes in EF and because previous findings suggest differences in the relation between PA and EF compared to older age groups^14–16^. Further, finding ways to promote EF as early as preschool age can have long-lasting and extensive positive effects on children’s lives, as EF present an important predictor for multiple positive life outcomes^3,4^. In this study, we implemented behavioral and questionnaire-based measures for both constructs: As a behavioral measure of PA, participants wore an accelerometer for 7 days. As a behavioral measure of EF, participants completed the German version of the EF Touch test battery. Parental questionnaires were also implemented to measure PA and EF. First, to ensure the validity of the implemented measures, we examined how behavioral and questionnaire-based measures of the constructs were related. For the other analyses, our focus lay on the behavioral measures of EF and PA, which promise a higher objectivity. Second, we hypothesized that accelerometer-assessed MVPA would be positively related to EF performance measured with the EF Touch, and we expected that controlling for hyperactive-impulsive behavior would improve the prediction of EF. Third, we hypothesized that hyperactive-impulsive behavior would moderate this relation between PA and EF.

Our finding that parent-reported PA and time spent in any PA intensity measured with the accelerometer were not significantly related contradicts the positive correlation reported in the original study validating the questionnaire^50^. Instead, our finding aligns with previous research showing differences in questionnaire- and accelerometer-derived PA levels^19^. This suggests that both might measure different aspects of PA: Questionnaire-based measures seem to be more suitable to provide information about the type of PA, while behavioral measures are more appropriate to reliably assess PA quantity. The latter are believed to be more robust^19^, for example because they capture the irregular nature of children’s everyday PA. Our finding that that parent-reported EF were significantly related to EF performance is in line with previous findings^44^. As hypothesized, if parents reported more EF-related problems, the children performed worse in the EF Touch.

In our study, accelerometer-assessed MVPA was negatively related to behavioral as well as questionnaire-based measures of EF—even when controlling for sociodemographic variables. This was in line with findings from Willoughby et al.^25^ who implemented the same measures. However, this result pattern contradicted both Willoughby et al.’s as well as our hypotheses, which based on previous findings supporting a positive association between PA and EF in preschoolers^17,55^ and older age groups^14–16^. To obtain a more detailed understanding of the relation between PA and EF, we also exploratively examined (1) the relation between MVPA and the separate EF components, and the association of (2) different accelerometer-assessed PA intensities as well as (3) parent-reported PA with total EF performance. A more in-depth discussion of these exploratory findings can be found in the Supplementary Material.

Our finding suggests that the association between everyday PA and EF in preschoolers might indeed differ from the well-established positive association in older age groups. However, more research is necessary to better understand the negative association observed in our study. In contrast, a meta-analysis of seven studies in early childhood^17^ suggested that PA was positively related to preschoolers’ EF. In particular, none of the included studies reported negative associations. It is important to consider that most of those studies implemented experimental study designs. Thus, they examined PA as induced or enhanced through specific interventions. In contrast, our study and Willoughby et al.’s study^25^ specifically investigated preschoolers’ everyday PA within their natural environment. Interventions often include more structured PA types that might be specifically beneficial for EF (e.g., with high cognitive demands^56^).

In contrast, an overall more physically active lifestyle may entail less structured types of PA that might be less favorable for EF development^23^. This might be especially the case in preschoolers, who are less likely compared to older age groups to undertake goal-directed and structured PA, for example for health-related reasons. Therefore, higher everyday PA levels in preschoolers might not necessarily be positively associated with EF. This is supported by a study that reported better EF in preschoolers that took part in structured PA, while objectively measured everyday PA was not related to EF^24^. Other studies in preschool samples that examined objectively measured everyday PA also reported no associations between MVPA and EF^23,57^, with only one study reporting a positive association^21^. Besides Willoughby et al.^25^, two other studies also reported negative associations between everyday MVPA and EF in preschoolers in samples very different to our Western and highly educated sample (i.e., low-income Brazil^22^, low-income, rural South Africa^23^). The diversity in the sample of the studies reporting negative associations between PA and EF suggests a high generalizability of the negative association observed in our study. Yet, possible moderators should be further examined to better understand this unexpected result pattern in preschoolers compared to older samples.

Willoughby et al.^25^ argued that preschoolers’ hyperactive-impulsive behavior might explain the unexpected negative relation between MVPA and EF: Preschoolers exhibiting higher hyperactive-impulsive behavior might engage in more PA^26^ and possibly in types of PA less favorable for EF development^23^. Previous research supports that children from clinical^32^ and non-clinical samples exhibiting more hyperactive-impulsive behavior^33^ showed higher objectively measured PA levels. This was not the case in our sample as parent-reported hyperactive-impulsive behavior was not related to time spent in any PA intensity. Other lines of research have argued that PA might be especially positively related to EF in children with high levels of hyperactive-impulsive behavior^43^, as they might compensate for lower EF capacities through higher PA levels^35^. Thus, another central aim of our study was to examine whether hyperactive-impulsive behavior moderates the relation between PA and EF. Our findings do not support Willoughby et al.’s^25^ argument that hyperactive-impulsive behavior explains the negative association between MVPA and EF. According to our findings, the relation between everyday PA and EF is neither stronger in children with high levels of hyperactive-impulsive behavior^35,43^ nor in those with low levels^25^.

Given the positive correlation between sedentary behavior and EF in our sample, we examined this association in addition to the pre-registered analyses (see Supplementary Material for results). The positive association between time spent sedentary and EF performance observed in our study contradicts findings from a review of 37 studies^43^: Here, sedentary behavior was negatively related to cognitive development in early childhood. However, the authors emphasized that different types of sedentary behavior (e.g., watching TV vs. reading) might have distinct effects on EF. Investigating children’s activities during sedentary time might be especially informative. In our study, parents had rather high educational levels and might have structured their children’s environment^43^ and sedentary activities more favorably for EF development (e.g., less screen time^58^), potentially explaining the positive association.

Despite the numerous advantages of observational study designs for the investigation of everyday PA, such designs do not allow any causal conclusions. Thus, the observed negative association between everyday MVPA and EF should not be interpreted as PA having a detrimental effect on EF. Rather, it is important to carefully examine how different types of PA are distinctly and potentially positively related to EF^24^. For example, PA incorporating mindfulness aspects (e.g., yoga^12^) or high cognitive load (e.g., team sports^56^) could be specifically beneficial for EF development. In our study, the employed questionnaire only contained limited information regarding the type of PA and its limited reliability further restricts the explanatory power. Future research should aim at combining quantitative and qualitative aspects of everyday PA with reliable measures. Our observational study design further restricts the interpretation of the positive association between sedentary behavior and EF, which might be reversed: Children with higher EF might be more interested in EF-promoting activities performed sedentarily, such as reading. Future studies could focus on examining the directionality of this association and further investigate specific sedentary activities^59^.

Our finding that parent-reported hyperactive-impulsive behavior was not related to PA levels contradicts previous findings^32,33^. Also, the non-significant moderating effect of hyperactive-impulsive behavior on the relation between PA and EF contradicts Burley et al.’s^33^ results: In their sample of 5- to 7-year-olds, hyperactive-impulsive behavior moderated the association between PA levels and EF performance, as the association increased with higher levels of hyperactivity. Following differences in the study designs should be considered when interpreting the different result patterns: (a) Burley et al. focused on PA during EF tasks, while we measured PA in preschoolers’ everyday life. (b) Burley et al. focused on hyperactivity, while we examined hyperactive-impulsive behavior to more extensively assess different aspects of ADHD symptomatology in preschoolers and their potential effect on the association between PA and EF. (c) Burley et al. specifically examined a sample of children with developmental difficulties and early ADHD symptomatology, while in our sample hyperactive-impulsive behavior was relatively low and showed limited variability. Thus, the possibility of observing an effect of hyperactive-impulsive behavior was limited in our sample. Future studies examining a possible moderating role of hyperactive-impulsive behavior should target samples with a wider variability in this construct and include preschoolers at risk for ADHD.

In our study, we focused on behavioral measures of PA and EF, which promise higher objectiveness and standardization. However, while accelerometers reliably assess children’s everyday PA, the EF Touch captures children’s EF performance at a selective time point. So, it does not reflect everyday EF performance. To mitigate this limitation in our study design and still ensure a high reliability and objectiveness, we suggest that future research should focus on repeatedly measuring children’s EF performance within their natural environment using ambulatory assessment^60^. As the EF Touch is a modular behavioral measure, it could be adapted and shortened to be used in future ambulatory assessment studies. This promises meaningful new insights into the relation between PA and EF in children’s everyday life.

Despite our efforts to mitigate potential sources of bias (e.g., diverse recruitment strategies, standardized administration of EF Touch), the exclusion of children with insufficient data potentially introduced bias: missing accelerometer data might have been more likely in children with higher hyperactive-impulsive behavior forgetting to wear the accelerometer or losing it. This directly affects the possibility of observing a moderating effect of hyperactive-impulsive behavior. Further, missing EF Touch data was more likely in children with lower EF capacities or with limited understanding of the tasks. This reduces the variability of EF performance in our sample, limiting the possibility of detecting associations to PA.

The composition of our sample presents another limitation: parents in our sample showed a rather high educational level, limiting the representativeness of our sample. In nearly all models conducted in this study, parental level of education was a significant positive predictor of EF performance. Further examining what aspects of the families’ environments or daily activities might be beneficial for EF development could help to better understand the potential additional effect of PA. Different indirect routes of promoting EF development exist^12^, and examining them collectively could yield more accurate insights into the effects of the promoting activities. For instance, PA might be especially beneficial for children receiving less other EF promotion.

## Conclusion

Contradicting our expectations, we found that time spent in MVPA was negatively related to German preschoolers’ EF performance. This supports findings from another study implementing the same EF measure in US preschoolers^25^. The present study represents the first study to investigate the proposition that hyperactive-impulsive behavior moderates the negative association between everyday PA and EF in preschoolers. Our findings do not support this proposition. The observational design of our study does not allow directional or causal inferences. Further, the rather low manifestation of hyperactive-impulsive behavior in our sample limited the possibility to detect a moderating effect. Future research should specifically include preschoolers at risk of ADHD and include more diverse samples concerning parental educational background. More research combining behavioral and questionnaire-based measures of PA is necessary to investigate the role of specific types of PA as well as different sedentary activities for EF development in preschool age.

## Supplementary Information

Supplementary Information.

## Data Availability

The datasets generated and/or analysed during the current study are not publicly available due to currently on-going data preparations but are available from the corresponding author on reasonable request. In the future, the datasets generated and/or analysed during the current study as well as the code used for analysis will be available in the OSF repository. Usage will be restricted to scientific purposes only.
