# Supplementary material for: Hyperactive-impulsive behavior does not moderate the association between executive function and physical activity in preschoolers
Source: Sci Rep. 2025 Apr 21;15:13792. doi: 10.1038/s41598-025-96791-0 (PMC12012208; doi:10.1038/s41598-025-96791-0)
Supplement: Supplementary file 1 — Supplementary Information. [file 41598_2025_96791_MOESM1_ESM.docx]

# Hyperactive-Impulsive Behavior Does Not Moderate the Association between Executive Function and Physical Activity in Preschoolers

# Supplementary Material

## Bivariate Associations between Assessed Variables

Unsurprisingly, we observed significant positive correlations between total EF Touch score and all EF components assessed with the EF Touch as well as between the single EF components (see *Supplementary Table S1*). Time spent in MVPA was related to more time spent in light PA and less sedentary behavior. Further, children’s age correlated significantly positively with total EF Touch score and all EF components, as well as sedentary behavior, but negatively with time spent in MVPA. Thus, MVPA levels decreased with age, while time spent sedentary increased. Parents’ answers in the BRIEF-P were positively correlated with hyperactive-impulsive behavior, implying that if parents reported lower EF for their child, they reported more hyperactive-impulsive behavior. Parents’ answers in the BRIEF-P as well as parent-reported hyperactive-impulsive behavior were both negatively related to total EF Touch score.

## Exploratory Analyses

### Data Analysis

In the exploratory analyses, we examined (1) how MVPA was related to the specific EF components assessed with the EF Touch (i.e., inhibition, working memory, shifting). Further, we examined (2) how time spent in different PA intensities besides MVPA (i.e., light, moderate, vigorous) was related to total EF Touch score. Since subjective and objective measures of PA were not significantly correlated, we also examined (3) if parent-reported PA was positively related to total EF Touch score. For this, we conducted linear regression models, controlling for age, child gender, BMI, parental level of education, and hyperactive-impulsive behavior: For Explorative Analysis 1, we used MVPA as predictor and conducted three separate models with (a) inhibition, (b) working memory, and (c) shifting as outcomes. For Explorative Analysis 2, we included proportional time spent in different PA intensities (i.e., light, moderate, vigorous) as predictors, and total EF Touch score as outcome. For Explorative Analysis 3, we used the parent-reported PA level as predictor and total EF Touch score as outcome.

**Supplementary Table S1**

Bivariate Correlations between Objective and Subjective Measures of Physical Activity and Executive Function

|  | 1. | 2. | 3. | 4. | 5. | 6. | 7. | 8. | 9. | 10. |
| --- | --- | --- | --- | --- | --- | --- | --- | --- | --- | --- |
| 1. Sedentary Behavior | - |  |  |  |  |  |  |  |  |  |
| 2. Light Physical Activity | **-.91 ***** | - |  |  |  |  |  |  |  |  |
| 3. MVPA | **-.80 ***** | **.48 ***** | - |  |  |  |  |  |  |  |
| 4. EF Touch – Total Score | **.30 *** | -.19 | **-.36 **** | - |  |  |  |  |  |  |
| 5. EF Touch – Inhibition | **.27 *** | -.18 | **-.31 *** | **.91 ***** | - |  |  |  |  |  |
| 6. EF Touch – Working Memory | .15 | -.06 | -.23 | **.73 ***** | **.41 ***** | - |  |  |  |  |
| 7. EF Touch – Shifting | .10 | -.06 | -.13 | **.69 ***** | **.54 ***** | **.38 **** | - |  |  |  |
| 8. Hyperactivity/Impulsivity | .03 | -.01 | -.05 | **-.25 *** | -.17 | -.08 | -.23 | - |  |  |
| 9. BRIEF-P | -.01 | .03 | -.03 | **-.25 *** | -.16 | **-.19 *** | -.20 | **.73 ***** | - |  |
| 10. Physical Activity Questionnaire | .10 | -.15 | .01 | -.01 | .03 | .06 | -.01 | -.02 | -.18 | - |
| 11. Age | **.25 *** | -.20 | **-.24 *** | **.69 ***** | **.64 ***** | **.45 ***** | **.64 ***** | -.16 | -.12 | .16 |

*Note*. * *p* < .05, ** *p* < .01, *** *p* < .001; Objective measures of physical activity refer to proportional time spent in specific physical activity intensity measured with the accelerometer (sedentary behavior, light physical activity, MVPA); MVPA – moderate-to-vigorous physical activity; EF Touch test battery was used as an objective measure of EF performance, accuracy scores were calculated for the different executive function components (total score, inhibition, working memory, shifting); BRIEF-P – parental questionnaire of executive function; age was calculated in months.

**Supplementary Table S2**

Relation between MVPA and Executive Function Components (Explorative Analysis 1)

|  | Inhibition | | | Working Memory | | | Shifting | | |
| --- | --- | --- | --- | --- | --- | --- | --- | --- | --- |
|  | Est. | CI | *p* | Est. | CI | *p* | Est. | CI | *p* |
| Intercept | **79.10** | **67.17 – 91.02** | **<0.001** | **48.58** | **35.97 – 61.19** | **<0.001** | **56.74** | **42.20 – 71.28** | **<0.001** |
| MVPA [in %] | -0.32 | -0.93 – 0.29 | 0.303 | -0.46 | -1.23 – 0.31 | 0.235 | -0.03 | -0.97 – 0.90 | 0.941 |
| Age | **0.73** | **0.43 – 1.04** | **<0.001** | **0.62** | **0.25 – 0.99** | **0.001** | **1.17** | **0.75 – 1.58** | **<0.001** |
| Gender (female) | 1.31 | -3.57 – 6.19 | 0.593 | -0.41 | -6.39 – 5.56 | 0.890 | 5.84 | -1.25 – 12.94 | 0.105 |
| Body Mass Index | -1.12 | -2.80 – 0.56 | 0.188 | 1.49 | -0.80 – 3.78 | 0.198 | -1.33 | -3.67 – 1.02 | 0.262 |
| Parental education | 0.87 | -1.76 – 3.50 | 0.511 | 2.29 | -0.47 – 5.05 | 0.102 | **3.40** | **0.16 – 6.65** | **0.040** |
| Hyperactivity/Impulsivity | -0.82 | -6.20 – 4.56 | 0.761 | -3.28 | -9.55 – 2.98 | 0.298 | -2.48 | -9.85 – 4.90 | 0.504 |
| *N* | 65 | | | 64 | | | 65 | | |
| R^2^ | 0.390 | | | 0.295 | | | 0.493 | | |
| Adjusted R^2^ | 0.327 | | | 0.221 | | | 0.440 | | |

*Note*. Multiple regression for Inhibition was calculated with Weighted Least Squares because of heteroscedasticity; MVPA – moderate-to-vigorous physical activity; Hyperactivity/impulsivity was assessed with a parental questionnaire; age was calculated in months.

### Results

Results of Explorative Analysis 1 are depicted in *Supplementary Table S2*. Examining the association between objectively measured MVPA and EF components in separate multiple regression models revealed no significant association for any EF component (inhibition: *β* = -0.32, *p* = .303; working memory *β* = -0.46, *p* = .235; shifting: *β* = -0.03, *p* = .941).

In Explorative Analysis 2, we inspected how time spent in different PA intensities was related to total EF Touch score in one multiple regression model, controlling for the same confounding variables as in Explorative Analysis 1 (see *Supplementary Table S3*). We found that no time spent in any PA intensity was significantly related to total EF Touch score (light PA: *β* = -0.14, *p* = .558; moderate PA: *β* = 0.07, *p* = .934; vigorous PA: *β* = -1.45, *p* = .173).

To investigate the association between parent-reported PA and EF in Explorative Analysis 3, we ran a separate multiple regression model with the same confounding variables (see *Supplementary Table S4*). No association between parent-reported PA and children’s total EF Touch score was evident (*β* = -0.25, *p* = .424). Only age was significantly related to higher EF (*β* = 0.92, *p* < .001).

**Supplementary Table S3**

Association between Time Spent in Different Physical Activity Intensities and Total EF Touch Score (Explorative Analysis 2)

|  | Est. | CI | *p* |
| --- | --- | --- | --- |
| Intercept | **60.25** | **51.55 – 68.94** | **<0.001** |
| Light Phyiscal Activity [in %] | -0.14 | -0.60 – 0.33 | 0.558 |
| Moderate Phyiscal Activity [in %] | 0.07 | -1.52 – 1.65 | 0.934 |
| Vigorous Physical Activity [in %] | -1.45 | -3.54 – 0.65 | 0.173 |
| Age | **0.71** | **0.46 – 0.96** | **<0.001** |
| Gender (female) | 0.83 | -3.45 – 5.10 | 0.699 |
| Body Mass Index | -0.42 | -1.77 – 0.94 | 0.540 |
| Parental Education | **2.40** | **0.44 – 4.36** | **0.018** |
| Hyperactivity/Impulsivity | -2.26 | -6.49 – 1.96 | 0.288 |

*Note*. *N* = 65, R^2^ = 0.569, adjusted R^2^ = 0.509. Physical activity was measured with an accelerometer; age was calculated in months.

**Supplementary Table S4**

Association between Parent-Reported Physical Activity and Total EF Touch Score (Explorative Analysis 3)

|  | Est. | CI | *p* |
| --- | --- | --- | --- |
| Intercept | **65.58** | **57.07 – 74.10** | **<0.001** |
| Parent-reported Physical Activity | -0.25 | -0.87 – 0.37 | 0.424 |
| Age | **0.92** | **0.68 – 1.15** | **<0.001** |
| Gender (female) | 2.90 | -0.80 – 6.60 | 0.122 |
| Body Mass Index | 0.35 | -1.13 – 1.83 | 0.639 |
| Parental Education | 1.08 | -0.81 – 2.97 | 0.256 |
| Hyperactivity/Impulsivity | -2.65 | -6.71 – 1.40 | 0.195 |

*Note. N* = 65, R^2^ = 0.588, adjusted R^2^ = 0.544. Multiple regression calculated with Weighted Least Squares; age was calculated in months.

### Discussion

Since previous research findings showed differential associations of EF components with PA as well as time spent in different PA intensities, we explored these separately in explorative analyses. First, we found no significant association between MVPA and any of the three EF components, in line with previous research^1,2^. This contradicts the findings by Willoughby et al.^3^ who observed a negative relation between MVPA and inhibition and a negative trend for working memory. Our findings suggest that investigating overall EF rather than separate EF components in preschoolers might yield more information, because in this age group the EF components are undergoing vast changes and follow different developmental trajectories^4,5^. Thus, preschoolers’ EF are possibly better described by one factor rather than separate components^6,7^.

Second, we investigated the association between time spent in different PA intensities and EF, as previous research revealed inconsistent findings depending on the examined PA intensity^2,8^. Here, we found that besides MVPA no time spent in any other PA intensity (i.e., light, moderate, vigorous) was significantly related to EF, even though negative trends were observable for all intensities. Third, since parent-reported and objectively measured PA were not related, we also examined the association between parent-reported PA and total EF Touch score. This analysis revealed no significant association. However, the PA questionnaire showed an extremely low reliability in our sample, limiting the interpretability of this analysis. Our study supports the well-established finding that the comparability between subjective and objective PA operationalizations is restricted^9^. Given the limited number of previous studies targeting preschoolers and the differences in implemented measurement methods, more research is necessary to obtain an overarching understanding of the relation between PA and EF in the developmentally sensitive period of preschool age.

## Additional Statistical Analysis – Sedentary Behavior

Since we observed a positive correlation between time spend sedentary and total EF (*r* = .31, *p* = .009), we also conducted a multiple regression model with sedentary behavior as predictor and total EF Touch score as outcome. Again, we controlled for age, child gender, BMI, parental level of education, and hyperactive-impulsive behavior. All control variables and the predictor were centered on the grand mean.

The multiple regression model examining sedentary behavior (see *Supplementary Table S5*) revealed a positive association between time spent sedentary and EF (*β* = 0.34, *p* = .013). More time spent in sedentary behavior was thus related to better total EF. Concerning the control variables, age (*β* = 0.39, *p* = .003) and parental level of education (*β* = 2.12, *p* = .031) were positively related to total EF.

**Supplementary Table S5**

Association between Sedentary Behavior and Total Executive Function Score

|  | Est. | CI | *p* |
| --- | --- | --- | --- |
| Intercept | 61.25 | 53.35 – 69.15 | **<0.001** |
| Sedentary Behavior [in %] | 0.27 | 0.03 – 0.51 | **0.028** |
| Age | 0.68 | 0.46 – 0.91 | **<0.001** |
| Gender (female) | 0.93 | -2.76 – 4.62 | 0.616 |
| Body Mass Index | -0.64 | -1.86 – 0.58 | 0.297 |
| Parental Education | 2.25 | 0.48 – 4.02 | **0.014** |
| Hyperactivity/Impulsivity | -1.86 | -5.66 – 1.95 | 0.333 |

*Note. N* = 65, R^2^ = 0.592, R^2^ = 0.550. Sedentary Behavior was measured with an accelerometer; age was calculated in months.

# References

1. Carson, V., Rahman, A. A. & Wiebe, S. A. Associations of subjectively and objectively measured sedentary behavior and physical activity with cognitive development in the early years. *Ment Health Phys Act* **13**, 1–8 (2017).

2. Mcneill, J. *et al.* Physical activity and modified organized sport among preschool children: Associations with cognitive and psychosocial health. *Ment Health Phys Act* (2018) doi:10.1016/j.mhpa.2018.07.001.

3. Willoughby, M. T., Wylie, A. C. & Catellier, D. J. Testing the association between physical activity and executive function skills in early childhood. *Early Child Res Q* **44**, 82–89 (2018).

4. Diamond, A. Executive functions. *Annu Rev Psychol* **64**, 135–168 (2013).

5. Garon, N., Bryson, S. E. & Smith, I. M. Executive Function in Preschoolers: A Review Using an Integrative Framework. *Psychol Bull* **134**, 31–60 (2008).

6. Karr, J. E. *et al.* The unity and diversity of executive functions: A systematic review and re-analysis of latent variable studies. *Psychol Bull* **144**, 1147–1185 (2018).

7. Ulitzka, B. *et al.* EF Touch – Testbatterie zur Erfassung der exekutiven Funktionen bei 3- bis 5- Jährigen deutschen Version. *Diagnostica* **69**, 182–193 (2023).

8. Vabø, K. B., Aadland, K. N., Howard, S. J. & Aadland, E. The multivariate physical activity signatures associated with self-regulation, executive function, and early academic learning in 3-5-year-old children. *Front Psychol* **13**, 842271 (2022).

9. Adamo, K. B., Prince, S. A., Tricco, A. C., Connor-Gorber, S. & Tremblay, M. A comparison of indirect versus direct measures for assessing physical activity in the pediatric population: A systematic review. *International Journal of Pediatric Obesity* **4**, 2–27 (2009).
